# Evaluation and Treatment Analysis of Air Quality Including Particulate Pollutants: A Case Study of Shandong Province, China

**DOI:** 10.3390/ijerph17249476

**Published:** 2020-12-17

**Authors:** Bowen Jiang, Yuangang Li, Weixin Yang

**Affiliations:** 1 College of Basic and Applied Sciences, Middle Tennessee State University, Murfreesboro, TN 37132, USA; bwjiang.tn@hotmail.com; 2 Business School, Dalian University of Foreign Languages, Dalian 116044, China; 3 Business School, University of Shanghai for Science and Technology, Shanghai 200093, China; iamywx@outlook.com

**Keywords:** particulate matter, air quality evaluation, air quality index, treatment analysis

## Abstract

At present, China’s air pollution and its treatment effect are issues of general concern in the academic circles. Based on the analysis of the development stages of air pollution in China and the development history of China’s air quality standards, we selected 17 cities of Shandong Province, China as the research objects. By expanding China’s existing Air Quality Index System, the air quality of six major pollutants including PM_2.5_ and PM_10_ in 17 cities from February 2017 to January 2020 is comprehensively evaluated. Then, with a forecast model, the air quality of the above cities in the absence of air pollution control policies since June 2018 was simulated. The results of the error test show that the model has a maximum error of 4.67% when simulating monthly assessment scores, and the maximum mean error of the four months is 3.17%. Through the comparison between the simulation results and the real evaluation results of air quality, we found that since June 2018, the air pollution control policies of six cities have achieved more than 10% improvement, while the air quality of the other 11 cities declined. The different characteristics of pollutants and the implementation of governance policies are perhaps the main reasons for the above differences. Finally, policy recommendations for the future air pollution control in Shandong and China were provided.

## 1. Introduction

Air pollution refers to the phenomenon that pollutants produced during natural evolution or human activities (such as dust, sulfides, nitric oxides, organic matters, etc.) and/or the secondary pollutants transformed from these pollutants cause harm to the human body and contaminate the living environment of human beings [1]. Since the reform and opening-up of China, as the economy rapidly expands, the problem of air pollution has also been growing quickly [2,3]. According to the examination results published by the Chinese Academy of Engineering and the Ministry of Environmental Protection in 2011, two-thirds of the cities in China failed to meet the national air quality standards [4]. Furthermore, the rapid pace of industrialization and urbanization in recent years has caused frequent changes in the air pollution problems as well as expanding pollution range [5]. Inhalable particles have become the primary air pollutant; after the stage of coal-smoke pollution and vehicle-emission pollution, China has entered a new stage of mixed pollution, which has the characteristics of multi-types of pollutants co-existing and various pollution sources interacting on different dimensions, processes and through different medium (refer to Table 1) [6,7,8,9,10].

At the same time, the air pollution assessment standards and control policies of China are not yet perfect and often lag behind the actual demand of the reality [11,12,13]. Although China has already issued a policy regarding industrial dust pollution control in 1956—“Decision of the State Council on Preventing Dust Hazards in Plants and Mining Companies”—its core was to improve the indoor working environment of the workers rather than control the air pollution [14]. It was not until 1982 that China developed its first Ambient Air Quality Standards (GB 3095-1982) [15], and it was not until 2012 that China included PM_2.5_ into monitoring as one of the main air pollutants (refer to Table 2) [15,16,17,18,19]. 

Even the latest Ambient Air Quality Standard (GB 3095-2012), which was developed in 2012 and officially implemented in 2016 and the Technical Regulation on Ambient Air Quality Index (HJ 633-2012), which defines the calculation methods of air quality evaluation scores, have some shortcomings that need improvement: (1) In these national standards, the first step in the calculation of air quality score is to calculate the sub-score of each pollutant indicator (i.e., the sub-score of six main air pollutants: PM_2.5_, PM_10_, CO, NO_2_, O_3_ and SO_2_) and then pick the largest indicator value as the final air quality score [19]. Therefore, the official air quality score of China actually only involves one air pollutant, which cannot objectively reflect the overall air quality. (2) When calculating the sub-indicator of the six main air pollutants, the national standards only requires the average value across a certain time period [19,20] and, therefore, cannot reflect the extreme value and fluctuations of the indicator during this time period. (3) Because there was a four-year gap between the development and the official implementation of the air quality standard, the upper limit of 24 h average PM_2.5_ score that was set to be 500 in this standard [19] cannot adapt to the reality of air quality when actually put into effect. Since 2016, there have been many cases in which the actual PM_2.5_ score of some cities in China has exceeded the upper limit of 500, i.e., the so-called “off-the-chart” [21,22]. Additionally, under the current air quality standards, the air quality evaluation of many cities has been simplified to the calculation and vertical/horizontal comparison of PM_2.5_ scores, while ignored the sub-scores of the other five main air pollutants. 

Therefore, we have extended the existing AQI Indicator System of China by introducing two sets of heterogeneous information apart from the average value—interval number and variance. Furthermore, through standardization of the heterogeneous information, the TOPSIS Method whose entropy coefficients are calculated by the Mahalanobis Distance has been adopted to comprehensively assess the air quality by taking the extreme value, average value and fluctuations of the six main air pollutants within the study period into full consideration. Meanwhile, the air quality scores of different cities in Shandong province in absence of air pollution control policies has also been simulated by the GM(1,1) Model based on sinusoidal function transformation and attempted to quantify the actual effect of the air pollution control policies by comparing the simulated scores with the actual air quality scores. 

In this paper, the 17 cities of Shandong province, including Ji’nan, Qingdao, Zibo, Zaozhuang, Dongying, Yantai, Weifang, Jining, Tai’an, Weihai, Rizhao, Laiwu, Linyi, Dezhou, Liaocheng, Binzhou and Heze, have been selected as research object. Shandong province has enjoyed booming economic growth in recent years, with its GDP rising from RMB 854.24 million in 2000 to RMB 7267.82 million in 2017, achieving an overall grow rate of 850.79% [23]. However, at the same time, Shandong province has also become one of the heavy-pollution areas in China. After Shandong province was listed as the key area for air pollution prevention and control in 2012 [24], in January 2013, out of these 17 cities, there were 16 cities marked by China’s Ministry of Environmental Protection as “heavily, severely and extremely seriously polluted”, in which 10 cities were marked as “severely polluted” and only Dongying was marked as “moderately polluted” [25]. This is quite surprising considering Shandong’s favorable geographic location. Shandong province enjoys a coastline of 3024.4 km (which is one-sixth of all the coastlines China has), over 20 natural harbors along its coastline and 296 islands close to the land [23]. Overall speaking, Shandong province is located in a smooth plain and enjoys plenty of rainfall thanks to the Temperate Continental Monsoon Climate [26,27]. Therefore, it is important to analyze and assess the air quality of cities in Shandong province, which has a rapid economic growth, advantageous geographic location and yet severe air pollution. It is also crucial to study the effectiveness of the air pollution control policies in order to provide a typical example of a China air quality study as well as anti-pollution policy effectiveness analysis to the academic circle. 

In recent years, scholars have paid close attention to the air quality of cities in Shandong. Wang et al. (2013) studied the impact of urbanization on the air sustainability in Shandong from 2005 to 2009 with a combination of Pressure–State–Response model and Balanced Scorecard. They showed that the urbanization and air environmental sustainability exhibited an upward trend in Shandong [28]. Zhu et al. (2015) used the eddy covariance technique to study the O_3_ concentration in the Northwest Shandong Plain. They found that solar radiation and temperature were the main factors affecting the O_3_ concentration [29]. Basing on the COPERT IV model and the vehicle age distribution in Shandong province from 2000 to 2014, Sun et al. (2016) studied the temporal trends and spatial distributions of air pollutants and greenhouse gases emitted by vehicles. They showed that the emissions of air pollutants had decreased, while greenhouse gas emissions had continued to increase in Shandong [30]. Yan et al. (2017) used the observation data of aerosol optical properties from December 2013 to May 2014 to research the air quality in Shandong. They indicated that local emissions were key sources of aerosols especially in Ji’nan, the provincial capital of Shandong, during heating period [31]. Li et al. (2017) analyzed hourly national air quality monitoring network data of normal pollutants at nine sites in Qingdao from 1 November 2015 to 31 January 2016. They argued that PM_2.5_ was the main pollutant in air during the above period. After investigating the pollution pathways and source distribution by the Hybrid Single-Particle Lagrangian Integrated Trajectory (HYSPLIT) model, they found that the west of Shanxi, south of Hebei, and west of Shandong accounted for 44.1% of the total air masses [32]. Zhang et al. (2018) used the samples collected simultaneously in one year at four sites in Shandong (Zibo, Zaozhuang, Qingdao and Ji’nan) to identify the source of PM_2.5_ and analyzed the related health risks. Basing on positive matrix factorization (PMF) model, they concluded that secondary formation, coal combustion and industry emissions were the main sources of PM_2.5_ in Shandong [33]. Zhang et al. (2018) adopted the Long-Range Energy Alternatives Planning System Model to analyze the coal consumption and emissions reduction in Shandong province in 2020. They concluded that improving air quality was the primary reason for controlling coal consumption, which should focused on four cities and three industries of Shandong [34].

The studies above on the air quality of different cities in Shandong province were still based on the existing air quality measurement standards of China, and many of them only considered main pollutants such as PM_2.5_ and PM_10_. Furthermore, many studies only focused on the air quality of a certain year or even a single month with a very short time span. Therefore, we have constructed an extended air quality indicator system covering the six main pollutants (SO_2_, NO_2_, CO, PM_10_, PM_2.5_ and O_3_) as specified in the national Air Quality Standards (GB 3095-2012 and HJ 633-2012). Since the above two national standards were only formally implemented in 2016, and the formulation of relevant local policies has a certain lag, we chose February 2017 to January 2020 as the research period to better evaluate the air pollution control effects in Shandong Province. This period is chosen because China’s latest Ambient Air Quality Standard (GB 3095-2012) was not official implemented until 2016. Based on this standard, Shandong Province formulated the “Measures for the Assessment of the Completion of Air Pollution Prevention and Control Targets and Tasks” in 2017 to measure and control air pollution [35]. Therefore, this period can reflect the actual effects of air pollution control in cities of Shandong according to the GB 3095-2012 standard.

The remainder of this paper is organized as follows. Section 2 provides the details of our methodologies. Section 3 presents our main empirical results and discusses the air pollution control policies launched by the local governments since June 2018 that may account for our results. Section 4 concludes the whole paper and provides policy recommendations.

## 2. Materials and Methods 

### 2.1. Indicator Construction and Standardization

We have adopted the daily average concentration value of the six main air pollutants and let the interval number be the difference between the biggest concentration value and the smallest value in the same month. The monthly average value and variance of this daily average concentration indicator have also been calculated. Among these three sets of heterogeneous information, the interval number $e_{ij}=\left[e_{ij}^{-},e_{ij}^{+}\right]\left(j\in C_{2}\right)$ has first been standardized to obtain its standardization form $x_{ij}$ as below:

(1) $$x_{ij}=\{\begin{array}{c} \left[\frac{e_{ij}^{-}}{\max(e_{.j}^{+})},\frac{e_{ij}^{+}}{\max(e_{.j}^{+})}\right],\;\;\;\;\;\;\;\;\;\;\;\;\;\;j\in C_{2}^{b}\\ \left[1-\frac{e_{ij}^{-}}{\max(e_{.j}^{+})},1-\frac{e_{ij}^{+}}{\max(e_{.j}^{+})}\right],\;\;\;\;\;\;\;j\in C_{2}^{c} \end{array}$$

in which $\max\left(e_{.j}^{+}\right)=max\left\{e_{ij}^{+}|i=1,2,\ldots ,m\right\}$.

Both the average and variance are real numbers and expressed by $e_{ij}=d_{ij}\left(j\in C_{1}\right)$. Their standardization form of $x_{ij}$ can be obtained by:

(2) $$x_{ij}=\{\begin{array}{ll} \frac{d_{ij}}{d_{maxj}}, & j\in C_{1}^{b};\\ 1-\frac{d_{ij}}{d_{maxj}}, & j\in C_{1}^{c}. \end{array}$$

in which $d_{maxj}=max\left\{d_{ij}|i=1,2,\ldots ,m\right\}$.

### 2.2. The TOPSIS Method with Entropy Weighted Coefficient Based on Mahalanobis Distance

After data processing, the TOPSIS Method, whose entropy coefficients are calculated by the Mahalanobis Distance, has been adopted to assess the air quality score of different cities in Shandong province. As a multi-target optimization evaluation method commonly used in environmental studies, the TOPSIS Method was first raised by C. L. Hwang and K. Yoon in 1981 [36]. It determines the relative evaluation scores of different objects by sorting them based on their proximity to the optimal value [37,38,39]. 

In the traditional TOPSIS method, there are two optimization targets. One is the positive optimal target (the best value), while the other is the negative optimal target (the worst value). The best evaluation object should be the one closest to the positive optimal target while farthest to the negative optimal target. If the assessment result of the TOPSIS model is expressed by a Proximity Function C, whose value is between 0 and 1, the closer a C value is to 1, the better assessment result it indicates, and vice versa [38,40]. The calculation steps are as below:

Suppose there are *m* objects and *n* evaluation principles. $W=\left(W_{1},W_{2},\ldots ,W_{j},\ldots W_{n}\right)$ represents the weight of each principle. First, the Characteristic Matrix *B* is constructed as:

(3) $$B=\left[\begin{array}{cc} \begin{array}{ccc} \begin{array}{c} x_{11}\\ x_{21}\\ \begin{array}{c} \vdots \\ x_{m1} \end{array} \end{array} & \begin{array}{c} \cdots \\ \cdots \\ \begin{array}{c} \vdots \\ \cdots \end{array} \end{array} & \begin{array}{c} x_{1j}\\ x_{2j}\\ \begin{array}{c} \vdots \\ x_{mj} \end{array} \end{array} \end{array} & \begin{array}{cc} \begin{array}{c} \cdots \\ \cdots \\ \begin{array}{c} \vdots \\ \cdots \end{array} \end{array} & \begin{array}{c} x_{1n}\\ x_{2n}\\ \begin{array}{c} \vdots \\ x_{mn} \end{array} \end{array} \end{array} \end{array}\right]\left(i=1,2,3,\cdots ,m;j=1,2,3,\cdots ,n\right)$$

in which $x_{mj}$ is the value of the $j^{th}$ indicator of the $m^{th}$ object. Then, the normalization vector will be calculated, and $r_{ij}$ will be the indicator after normalization. The normalization method is explained as in Equation (4):

(4) $$r_{ij}=\frac{x_{ij}-{\left(x_{ij}\right)}_{min}}{{\left(x_{ij}\right)}_{max}-{\left(x_{ij}\right)}_{min}},i=1,2,\cdots ,m;j=1,2,\cdots ,n$$

Then, the Weight Normalization Vector $v_{ij}$ is constructed as below:

(5) $$v_{ij}=w_{j}r_{ij},i=1,2,\cdots ,m;j=1,2,\cdots ,n$$

Hence, the positive optimal solution $v^{*}$ and the negative optimal solution $v^{-}$ can be calculated. Here, $v^{*}$ is the maximum value in the matrix array after normalization, while $v^{-}$ is the minimum value in the matrix array after normalization, as shown in Equation (6).

(6) $$\{\begin{array}{c} v^{*}=(max_{i}v_{ij}|j\in J_{1}),(min_{i}v_{ij}|j\in J_{2}),|i=1,2,\cdots ,m\\ v^{-}=(min_{i}v_{ij}|j\in J_{1}),(max_{i}v_{ij}|j\in J_{2}),|i=1,2,\cdots ,m \end{array}$$

Based on the traditional TOPSIS Method, the TOPSIS Method whose Entropy Weighted Coefficient is calculated based on the Mahalanobis Distance will be further introduced. Under the traditional TOPSIS Method, the calculation of the distance between a certain object’s indicator evaluation result and its positive optimal target is based on the two-dimensional distance. The commonly used calculation methods in academic literature include the Euclidean Distance Method [41,42,43] and the Minkowski Distance Method [44,45,46]. The Minkowski Distance Method measures the differences between unified indicators of multiple decision objects by calculating the distance between vectors. The Euclidean Distance Method is the most common method for two-dimensional distance calculation. It is also the special case when the dimension parameter in the Minkowski Distance Method equals two [47,48,49]. 

The traditional TOPSIS Method has some shortcomings that need improvement [50,51]: (1) When there are large numbers of indicators, the importance of various indicators needs to be further differentiated. (2) There is a risk of inverted order, i.e., the increase or decrease in the number of decision objects would impact data standardization, the positive optimal solution and the negative optimal solution, which would further impact the calculation of distance. (3) There are still some controversies about the order or ranking stability under the Euclidean Distance Method. Therefore, considering the heterogeneous information indicators constructed around the six air pollutants, we adopted the Mahalanobis Distance Method for distance calculation. The Mahalanobis Distance Method is also a calculation method for distance between vectors. Its main advantage is that it is not impacted by the choice of dimensions and can eliminate the correlation between variables [52,53,54]. A Positive Optimal Solution vector with the optimal solutions of various indicators has been composed, and a Negative Optimal Solution vector with the worst solutions has also been constructed. Moreover, a Decision Vector made up of the evaluation values of various indicators of each decision object has been designated [55,56]. It is worth noticing that when using the Mahalanobis Distance Method to calculate distances, we need to make sure the covariance matrix is positive definite, i.e., each element in the covariance matrix is the covariance in each vector element. The detailed steps are as follows: 

Let $S^{+}$ be the Positive Optimal Vector comprised optimal values, and $S^{-}$ be the Negative Optimal Vector comprised of the worst values. $A_{i}$ is the Decision Vector of the $i^{th}$ decision object. The distance between $A_{i}$ and the Positive Optimal Vector $S^{+}$, and the distance between $A_{i}$ and the Negative Optimal Vector $S^{-}$ are, respectively, expressed in Equation (7) below:

(7) $$\{\begin{array}{c} d\left(A_{i},S^{+}\right)={\left[{\left(v_{i}-S^{+}\right)}^{T}\sum -1\left(v_{i}-S^{+}\right)\right]}^{\frac{1}{2}}\;\\ d\left(A_{i},S^{-}\right)={\left[{\left(v_{i}-S^{-}\right)}^{T}\sum -1\left(v_{i}-S^{-}\right)\right]}^{\frac{1}{2}} \end{array}$$

in which $d\left(A_{i},S^{+}\right)$ represents the distance to the positive optimal solution, while $d\left(A_{i},S^{-}\right)$ is the distance to the negative optimal solution, and ∑−1 is a symmetric positive definite matrix. Based on the distance to the positive and negative optimal solution, the Proximity Score $C^{*}$ can be obtained as shown in Equation (8) below.

(8) $$C_{i}^{*}=\frac{d\left(A_{i},S^{+}\right)}{\left(d\left(A_{i},S^{+}\right)+d\left(A_{i},S^{-}\right)\right)},i=1,2,\cdots ,m$$

In this equation, the bigger the Proximity Score $C^{*}$ is, the better air quality it represents. The smaller Proximity Score $C^{*}$ is, the worse air quality a city has [57]. 

Through this TOPSIS Method with Entropy Weighted Coefficient calculated based on the Mahalanobis Distance, this improved TOPSIS Method has been involved into final decision-making to reduce the bias in calculation results due to shortcomings of the model.

## 3. Results and Policy Analysis

### 3.1. The Calculation Results

With help of the methodologies introduced in Part 2 and the MATLAB algorithm (the software of MathWorks, Inc. Natick, MA, USA. Version: r2017b) in Appendix A, and based on the official daily air pollutant data (PM_2.5_, PM_10_, CO, NO_2_, O_3_ and SO_2_) from February 2017 to January 2020 published by the Data Center of Ministry of Ecology and Environment of the People’s Republic of China [58], the air quality assessment scores of the 17 cities of Shandong province during the study period have been calculated (as shown in Table A3, Table A4, Table A5 and Table A6 in Appendix D).

During our study period, Shandong province launched large numbers of air pollution control policies in 2018: The local government of Shandong Province issued “Action Plan of Shandong Province for Air Pollution Prevention and Control 2013–2020 (Phase III, 2018–2020)”. It has announced the target of achieving a 35% improvement in air quality by 2020 (compared with that of 2015), as well as five major measures including energy structure and industrial structure upgrade, pollution prevention and control in key industries, comprehensive regulation on dust pollution, vehicle emission pollution control and construction of an ecological green belt [59]. 

For quantifying the effect of these air pollution control policies, the GM(1,1) Model based on Sinusoidal Function Transformation and the MATLAB algorithm (refer to Appendix B) have been adopted to simulate the dynamic changes in air quality in the absence of pollution control policies in different cities of Shandong Province [60]. The simulated air quality scores has also been compared with the actual scores in Table A3, Table A4, Table A5 and Table A6 in Appendix D (especially the actual air quality scores after June 2018 or since the pollution control policies took effect) to quantify the effectiveness of different cities’ pollution control policies.

Through calculations above, the simulated air quality scores in the absence of pollution control policies in different cities of Shandong province have been obtained (refer to Appendix C). 

**Figure 1:**
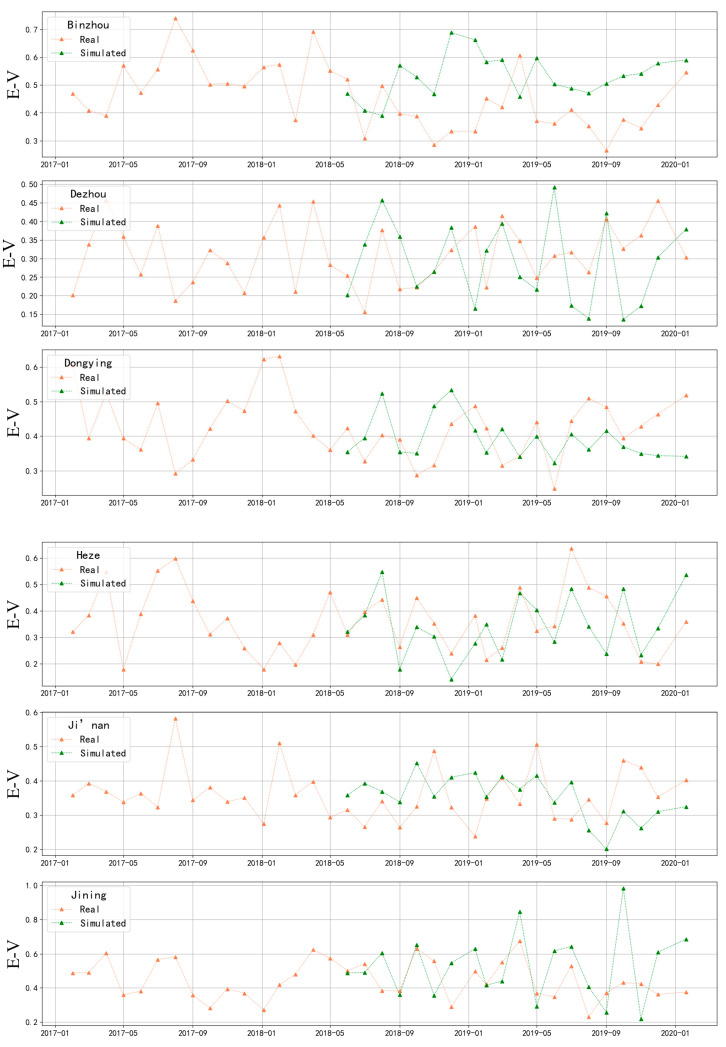

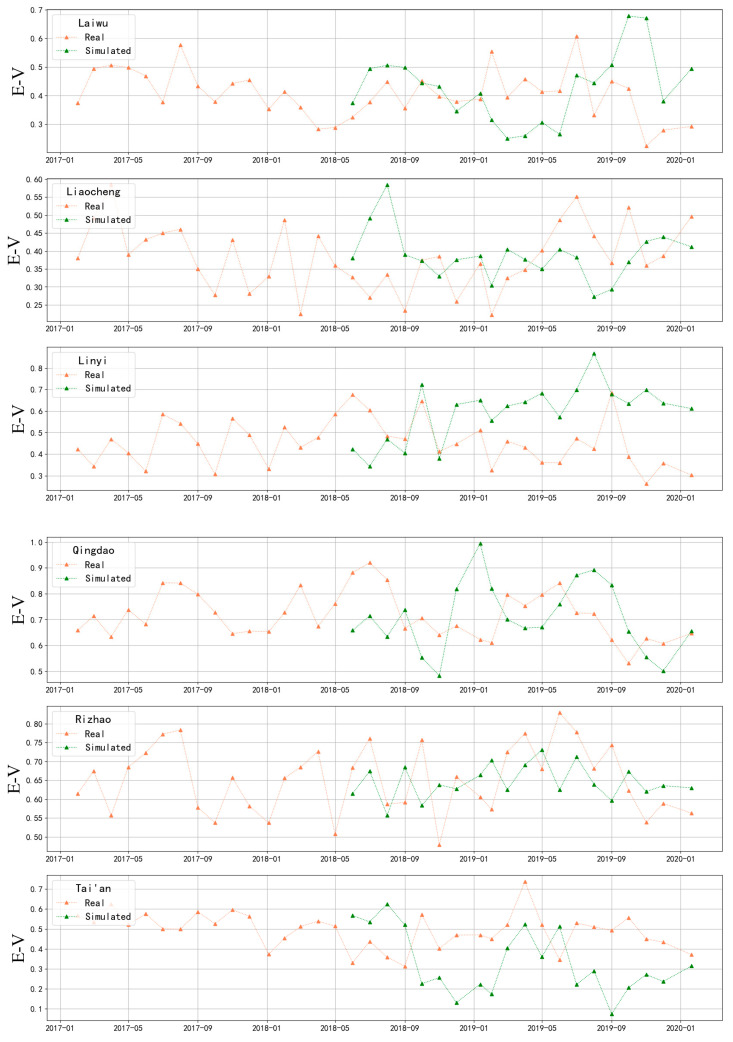

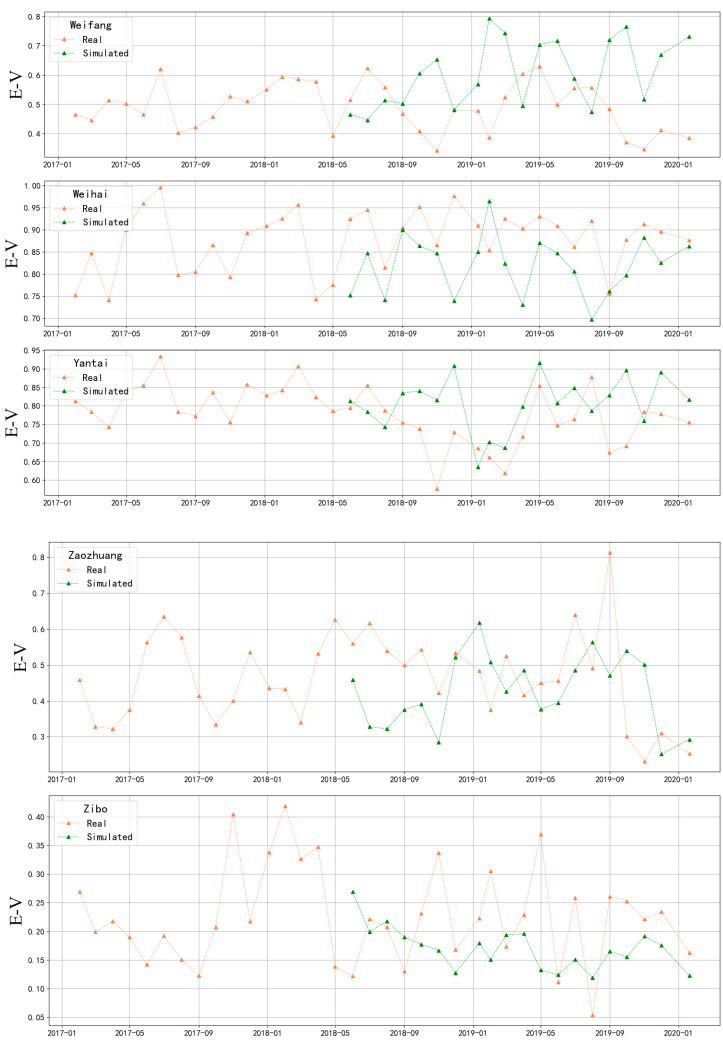
Comparison between the values of simulated air quality scores and actual air quality scores of cities in Shandong province.

Thus, the comparison between air quality assessment score simulated by the GM(1,1) Model based on Sinusoidal Function Transformation and real air quality of cities in Shandong province has been achieved (refer to Figure 1).

Because the governance policies of Shandong mainly began in June 2018 in the research period, we used the real assessment scores from February 2017 to May 2018 in the above calculation results to test the error of the model. Based on the real air quality assessment scores of cities in Shandong from February 2017 to January 2018, we first obtained the simulated air quality assessment scores from February 2018 to May 2018 (please refer to Table A7 in Appendix E). Then, the error of the model was obtained by comparing the simulated values with the real assessment scores of air quality from February 2018 to May 2018 (please refer to Table A8 in Appendix E). The results show that the model has a maximum error of 4.67% when simulating monthly assessment scores, and the maximum mean error of the four months is 3.17%.

By comparing the simulated air quality scores with the actual scores, it has been found that different impacts of the air pollution control policies that were implemented since June 2018 on different cities of Shandong Province. There are distinct differences in terms of policy effectiveness.

### 3.2. The Improvements of Air Quality

The air quality scores of six cities including Ji’nan, Dezhou, Liaocheng, Weihai, Binzhou and Heze have seen clear improvements when comparing the ending value (Jan 2020) with the beginning value (Feb 2017). Among these cities, Dezhou has experienced the biggest improvement of 50.12% in air quality, while Liaocheng has also improved its air quality by 30.53%; the rest of the cities (Ji’nan, Weihai, Binzhou and Heze) have all improved their air quality by over 12%. It is worth noticing that among these six cities, five of them are located on the transmission path of air pollution in the Beijing–Tianjin–Hebei Region (Ji’nan, Dezhou, Liaocheng, Binzhou and Heze), and therefore were listed among the “26+2” key cities in the campaign of air pollution prevention and control in the Beijing–Tianjin–Hebei Region [61]. The list was named as “26+2” because it included two special municipalities directly under the central government (Beijing and Tianjin), and another 26 cities under the four provinces of Hebei, Henan, Shandong and Shanxi. This campaign was jointly implemented and seriously emphasized by China’s Ministry of Environmental Protection, the National Development and Reform Commission, Ministry of Finance, Bureau of Energy and the local governments of Beijing, Tianjin, Hebei, Henan, Shandong and Shanxi [62]. 

The effectiveness of the air pollution control policies of Dezhou is due to the local government’s determination and commitment. Apart from the centralized pollution control measures of Shandong province, Dezhou local government took the lead in passing the “Regulations on Air Pollution Prevention and Control of Dezhou City”. This was the first local air pollution prevention and control regulation among all cities of Shandong province, as well as the first government regulation issued after Dezhou government obtained local legislative power. This regulation has specified five principles of air pollution prevention, including ecology first, prevention and treatment integrated, comprehensive management, government-led and public participation. It has also determined policy initiatives including “reducing coal usage, preventing dust pollution, controlling vehicle numbers, deodorization, and increasing greenbelts” based on the actual conditions of the city [63]. In 2019, the city has planned to eliminate all coal-fired boilers of 35 steam tons and below. It has also taken the lead in promoting clean energy heating projects, encouraged and enforced the use of low-pollution clean coal in the entire city and completed the “Coal-to-Gas and Coal-to-Electricity” transformation of 67,000 households. It has also issued the “Dust Control Order” within the central urban area, stopped all construction earthwork operations during the heating season and eliminated all yellow-label vehicles (i.e., heavy-polluting vehicles) and old cars. The pass rate of spot check on automobile gasoline and diesel oil has reached 97.9%, and the proportion of clean energy and new energy buses in the central urban area has reached over 97%. The Dezhou local government has also completed the construction of level-three oil and gas recovery equipment in all gas stations in its urban area and counties and ordered 191 companies with above-standard VOC (Volatile Organic Compounds) emissions to take measures, in which all petrochemical companies have taken pollution control actions. At the same time, 4026 auto repair companies in the city were comprehensively inspected and rectified, and open-air painting operations were prohibited. The “Three-Year Greening” Project and the “Green Dezhou, Ecological City” Initiative were implemented, with an annual afforestation of over 200 thousand Mu. The local government has also started the “Grand Canal Ecological Forest Project” [64]. Above governance policies of Dezhou city have achieved positive results. There have been obvious improvements in its overall air quality since 2019. Consistent with our evaluation results, the official statistics of China’s Ministry of Environmental Protection showed that the improvement of air quality of Dezhou city in autumn and winter 2019–2020 is obvious among the “2+26” cities [65]. 

As the provincial capital of Shandong Province, Ji’nan has improved its air quality by 12.28% when comparing its ending score with the beginning score, and by January 2020, its air quality score had improved by 27.69% when compared with that of June 2018 when the air pollution control policies were first implemented. Since June 2018, the local government of Ji’nan has initiated the development of the “Air Pollution Prevention and Control Action Plan (Phase III)” based on the centralized pollution control measures of Shandong province, which was officially published in December that year. According to this Action Plan, Ji’nan will analyze different sources of air pollution in high detail and construct the first database of urban pollution sources and their components among all cities in Shandong [66]. Based on scientific analysis on the sources of atmospheric pollutants, Ji’nan eliminated all coal-fired boilers of 35 tons and below in its city from 2018 to 2019, becoming the first provincial capital to complete this task and was selected to be the pilot city for the clean heating project in Northern China during winters [67]. Meanwhile, Ji’nan has completed the ultra-low emission transformation of 94 coal-fired boilers with a capacity of more than 35 tons and initiated the “Coal-to-Gas and Coal-to-Electricity” transformation of 110,000 households in the city [68]. From April 2017, the Shandong Provincial Government officially implemented transformation measures on one of the major polluters in Ji’nan, the Ji’nan Iron and Steel General Plant [69]. At the same time, Ji’nan also completed the relocation of 54 heavy-pollution companies in its old industrial park in the eastern city and completed the cleanup and rectification of 7190 companies with poor pollution management. Regarding the serious vehicle emissions, which far exceeded the standards, the local government of Ji’nan has steadily moved forward the work on oil product upgrading and construction of level-three oil and gas recovery equipment. It has prohibited the sale of substandard gasoline and diesel products across the city, installed level-three oil and gas recovery equipment in all gas stations and transformed 187 gas stations in the city in order to effectively control the increase in VOC emissions [68]. The effect of above air pollution control policies has been reflected in the official statistics of China’s Ministry of Environmental Protection: in January 2020, the improvement rate of Ji’nan’s air quality ranked the 13th among the “2+26” cities [70]. 

Although the air quality of Liaocheng, Binzhou and Heze did not reach an ideal level in our study period (their air quality scores of January 2020 were 0.4964, 0.5453 and 0.3595, and ranked seventh, fifth and twelfth among cities of Shandong province, respectively), their air quality scores have all seen significant improvements (by 30.53, 16.20 and 12.12%, respectively) when compared with their beginning level in February 2017. Apart from strictly follow the air pollution control policies of Shandong Province and the national centralized pollution control measures for “2+26” cities, the above three cities have taken more targeted measures according to their own characteristics: (1) Liaocheng government encouraged reduction in coal consumption and the use of substitutes and split new projects with high coal consumption to different counties (districts), development zones and key enterprises, which helped Liaocheng to have achieved a total reduction of 1.27 million tons in coal consumption in 2019 when compared with 2014. Liaocheng government has also transformed all coal-fired boilers of more than 20 tons to high-efficiency pulverized coal boilers. All coal-fired boilers of over 20 tons are transformed to be using clean energy such as electricity, gas and biomass energy. All coal-fired boilers of 4 tons and below are transformed to be electricity- or gas-based, whose installation and operation will be monitored by the local government and the environmental protection department through an all-weather online monitoring network. In the winter heating season, all pharmaceutical and pesticide raw materials companies are required to stop production, and manufacturers of electrolytic aluminum and alumina are subject to an at least 30% cut in production [71]. (2) Binzhou government directly eliminated electrolytic aluminum companies and small coal-fired boilers. By the end of July 2019, it has shut down a total electrolytic aluminum capacity of 2.68 million tons. By the end of October 2019, it has eliminated 3205 coal-fired boilers with a capacity of 10 tons and below within its jurisdiction and required that all gas stations whose annual gasoline sales volume is over 5000 tons install online monitoring equipment for oil and gas recovery. Meanwhile, it has implemented the staggering transportation peak policy for key enterprises, which requires enterprises, such as Weiqiao Pioneering Group Co., Datang Corp. (Binzhou), Huaneng Zhanhua Power Plant, etc., to reduce their volume of bulk cargo by road transportation by over 50% within certain periods of time according to the requirements of the environmental protection department [72]. (3) On the basis of analyzing the sources of atmospheric particulate matter and summarizing the list of pollution sources, Heze government focused on cleaning up and reorganizing the companies with poor pollution management. By the end of 2019, it had banned 3691 companies and shut down 622 companies. In view of its city characteristic of serious vehicle emissions in the urban area, Heze government has enforced the usage of National-VI standards by new diesel vehicles and banned the sales of ordinary diesel and gasoline and diesel fuels below the National-VI standards. In 2019, Heze government had also equipped itself with fixed and mobile remote sensing monitoring equipment, which has connected with the network of China’s Ministry of Ecology and Environment and the Environmental Protection Bureau of Shandong Province [73]. 

Weihai is the only city among the six cities in Shandong that has achieved improvement in air quality but not been listed among the “2+26” key monitored cities. Weihai is located far away from the Beijing–Tianjin–Hebei air pollution transmission path, with its north, east and south sides close to the Yellow Sea [74], which provides an advantageous geographical condition for its air quality. Furthermore, since 2017, Weihai has continuously strengthened its air pollution control policies: completed ultra-low emission transformation of 53 coal-fired boilers, dismantled 78 coal-fired small boilers in the city, managed 16 projects on VOCs emission governance and control and completed the construction of level-three oil and gas recovery equipment in 134 gas stations [75]. Therefore, the air quality evaluation score of Weihai has been higher than 0.7400 throughout the study period, ranking top among the cities of Shandong province. Weihai’s air quality score in January 2020 was 0.8767, ranking first in Shandong province. This was further confirmed by the official statistics: the Shandong Provincial Government announced in 2018 that Weihai’s air quality has kept ranking first in the province, and therefore, it decided to grant Weihai a recognition award with 10 million RMB [76].

### 3.3. The Declines of Air Quality

The air quality of 11 cities of Shandong Province (Qingdao, Yantai, Rizhao, Dongying, Weifang, Laiwu, Jining, Linyi, Tai’an, Zibo and Zaozhuang) has decreased by varying degrees when comparing the ending value (2020.01) with the beginning value (2017.02). The air pollution control policies did not show much effect in those cities, more specifically: 

The air quality score of Qingdao, Yantai and Rizhao did not experience much change within in the study period when comparing their ending score with the beginning one—reduced by 1.75, 7.06 and 8.45%, respectively. The common feature of these three cities is that they are located on the eastern coast of Shandong Province. The overall air quality is good, but the urban air quality shows a clear seasonal pattern, with heavy air pollution during winter and spring. This is mainly due to the low temperature in winter and urban heating. The dramatic increase in coal consumption has also brought large amounts of coal dust pollution. During spring, the climate is dry with little rainfall, and therefore not easy for dust to drift away [77]. Furthermore, the sandstorm weather of Northern China has also brought negative impacts. According to the statistics of the Institute of Atmospheric Physics of the Chinese Academy of Sciences, 25% of the PM_2.5_ pollution in Rizhao were transmitted from the outside [78]. These pollution patterns have offset the efforts of governance policies to a certain extent, thus causing a lower air quality of these three cities in January 2020 when compared with that of June 2018 when the centralized air pollution control policies were implemented. 

The air quality of the eight cities including Dongying, Weifang, Laiwu, Jining, Linyi, Tai’an, Zibo and Zaozhuang has shown obvious deterioration when comparing the ending score with the beginning one. The main reasons are: The above cities are mainly located in the south-central hinterland of Shandong province, susceptible to air pollution transmitted from outside, especially during autumn and winter seasons when they are greatly affected by the northwest wind and heating season coal pollution [79]. Moreover, the air pollution control policies of these cities are not centralized with poor enforcement. For example, Jining did not adopt further prevention policies after achieving initial results in air pollution control, which led to a rebound of air pollution. In the first quarter of 2019 alone, 19 units in the city were criticized by the provincial government due to poor air pollution control [80]. As of January 2020, there have already been 52 batches of air pollution prevention and control issues publicly exposed by the Jining News Network alone [81]. According to the inspection result of pollution control policy implementation and environmental protection, Weifang did not expand the scope of air pollution supervision and monitoring from the city center to the entire city and its counties until 2018 [82]. During the inspection by Shandong Provincial Environmental Protection Inspectorate on Dongying, many pollution cases of have been found [83]. On August 29, 2019, the Ministry of Ecology and Environment organized an investigation team to conduct an unannounced visit to Linyi. It was found that the air pollution control work there usually does nothing. However, when it comes to assessing accountability, Linyi always rushes for quick results and passes many “one size fits all” measures [84]. Furthermore, Tai’an has been publicly interviewed by the Shangdong Provincial Government, specially inspected by the Central Ecological Environmental Protection Supervision Office, and interviewed by the Ministry of Ecology and Environment due to the obvious deterioration of air quality since 2019 [85]. 

## 4. Conclusions

Through extended AQI Indicator System of China, we have calculated and evaluated the air quality score of cities in Shandong province from February 2017 to January 2020 by using the TOPSIS Method whose entropy coefficients are calculated by the Mahalanobis Distance. The air quality scores of different cities in Shandong province since June 2018 in the absence of air pollution control policies have also been simulated based on the GM(1,1) Model based on Sinusoidal Function Transformation. Our findings are that the air pollution control policies implemented since June 2018 have shown distinctly different impacts in different cities with huge disparity. The air quality of six cities, including Ji’nan, Dezhou, Liaocheng, Weihai, Binzhou and Heze, has shown improvement of over 12% during the study period. However, mainly due to the unique characteristics of their air pollutants and weak enforcement of pollution control policies, the air quality of the other 11 cities has experienced a decrease in different degrees when comparing their ending score with the beginning one. Based on the findings above, policy recommendations for Shandong province have been proposed as below: 

First of all, Shandong should integrate the air pollution prevention and control measures of the “2+26” key cities to achieve overall planning of pollution control. Among the “2+26” key cities, those under Shandong province (such as Ji’nan, Dezhou, Liaocheng, Binzhou and Heze) have achieved significant improvements in air quality under the pollution control measures of the central government. However, considering the large disparity in policy effectiveness among cities of Shandong, the government still needs overall planning and coordination of the pollution control policies and to ensure the effectiveness of these policies in order to further improve the air quality of all cities. 

Moreover, the government should fully consider the differences in air pollution between coastal cities (Weihai, Yantai and Qingdao) and the inland cities and develop targeted pollution control policies. In terms of the characteristics of air pollution, there are significant differences in air pollution between the coastal cities in eastern Shandong and the inland cities in the central and western regions of the province, and there are also considerable gaps in air quality. Therefore, Shandong need to develop targeted pollution control policies based on the fact that inland cities have large proportions of imported pollution from the outside and the severe air pollution due to coal burning in the heating season cannot dissipate easily in the short term. In this respect, the policies of Liaocheng such as coal consumption reduction and substitution, high-efficiency pulverized coal boiler transformation and “Coal-to-Gas and Coal-to-Electricity” transformations [71] have achieved outstanding results and are recommended to be promoted based on the characteristics of specific cities. 

Last but not the least, the government should strengthen the implementation of pollution control policies and improve the environmental inspection system. The decline in air quality of some cities in Shandong province is mainly due to poor implementation and enforcement of pollution control policies, which has resulted in the many problems discovered during the provincial and national environmental protection inspections. Therefore, in future, air pollution control campaigns, apart from policy design and development, Shandong should also pay close attention to the implementation and enforcement of these policies, with help of the continuously improved environmental supervision system and other supervision and inspection systems.

## Figures and Tables

**Table 1:** The development stages of air pollution in China.

	1949–1990	1991–2000	2001–2009	2010–Present
Main Pollutants	SO_2_, TSP, PM_10_	SO_2_, NO_x_, TSP, PM_10_	SO_2_, PM_10_, PM_2.5_, NO_x_, VOCs, NH_3_	PM_2.5_, PM_10_, NO_2_, CO, O_3_, SO_2_, VOCs, NH_3_
Pollution Types	Coal ash	Coal ash, acid rain, particulate pollutant	particulate pollutant, coal ash, photochemical pollution, dust-haze	dust-haze, fine particulate matter, photochemical pollution, ozone, coal ash
Pollution Sources	Coal burning, industrial pollution	Coal burning, industrial pollution, dust	Coal burning, industrial pollution, dust, motor vehicle pollution	Coal burning, industrial pollution, dust, motor vehicle pollution, soil dust, secondary inorganic aerosol
Polluted Regions	Major industrial bases	Industrial bases, some cities	Many cities	Most cities of the country
Occurrence Frequency	Seldom	Sometimes	Often	Frequently

**Table 2:** Development history of China’s air quality standards.

Year	Standard	Key Points
1982	Ambient Air Quality Standards (GB 3095-1982)	Defined the main air pollutants to be monitored as total suspended particulates (TSP), floating dust, SO_2_, CO, O_3_ and oxynitride (measured in NO_2_) Regulated the monitoring methods of these six pollutants
1988	The Maximum Allowable Concentration of Atmospheric Pollutants for Crops (GB 9137-88)	Classified different crops based on their sensitivity to air pollution into 3 categories—Sensitive Crops, Medium Sensitive Crops, Resistant Crops Regulated the maximum allowable concentration of SO_2_ and fluoride to protect vegetables, fruit trees, mulberry tea, pasture and other critical cash crops
1996	Ambient Air Quality Standards (GB 3095-1996)	Added definitions of 14 terms, including total suspended particulates (TSP), inhalable particles, etc. Added pollutant items such as PM_10_, and adjusted the measuring time and concentration limits Added regulations on the effectiveness of statistical data measurement of various pollutants
2000	Ambient Air Quality Standards (GB 3095-1996) (Revised Edition)	Dropped the indicator for oxynitride (NO_X_) Relaxed the regulations on the Level 2 concentration limits of nitrogen dioxide (NO_2_) Relaxed the regulations on the Level 1 and Level 2 hourly average concentration limits of ozone (O_3_)
2012	Ambient Air Quality Standards (GB 3095-2012) (Effective since January 2016)	Added regulations on the concentration limits of PM_2.5_ pollutants and 8 h average concentration limits of O_3_ Adjusted the concentration limits of pollutants including PM_10_ and NO_2_

## Appendix A. MATLAB Algorithm for the TOPSIS Method with Entropy Weighted Coefficient Based on Mahalanobis Distance

**Algorithm A1**
1: [n,m]=size(x); 2: zh=zeros(1,m); 3: d1=zeros(1,n); 4: d2=zeros(1,n); 5: c=zeros(1,n); 6: **for** i=1:m 7: **for** j=1:n 8: zh(i)=zh(i)+x(j,i)^2; 9: **end** 10: **end** 11: **for** i=1:m 12: **for** j=1:n 13: x(j,i)=x(j,i)/sqrt( zh(i)); 14: **end** 15: **end** 16: xx=min(x); dd=max(x); 17: **for** i=1:n 18: **for** j=1:m 19: d1(i)=d1(i)+(x(i,j)-xx(j))^2; 20: **end** 21: d1(i)=sqrt(d1(i)); 22: **end** 23: **for** i=1:n 24: **for** j=1:m 25: d2(i)=d2(i)+(x(i,j)-dd(j))^2; 26: **end** 27: d2(i)=sqrt(d2(i)); 28: **end** 29: **for** i=1:n 30: c(i)=d1(i)/(d2(i)+d1(i)); 31: **end**

## Appendix B. The GM(1,1) Model Based on Sinusoidal Function Transformation and MATLAB Algorithm

### Appendix B.1. The GM(1,1) Model Based on Sinusoidal Function Transformation

We have adopted the GM(1,1) Model transformed from the sinusoidal function sinx$\left(x\in \left(2b\pi -\frac{\pi }{2},2b\pi +\frac{\pi }{2}\right)\right)$ to simulate the air quality scores of different cities in Shandong province in absence of air pollution control policies. The detailed steps are as follows:

(1) Sort the raw data and build an incremental sequence $A^{\left(0\right)}$:

(A1) $$A^{\left(0\right)}=\left\{a^{\left(0\right)}\left(1\right),a^{\left(0\right)}\left(2\right),\cdots ,a^{\left(0\right)}\left(n\right)\right\}$$

in which $a^{\left(0\right)}\left(i\right)>0,i=1,2,\cdots ,n$.

(2) Transform the original sequence so that it is in a monotonically decreasing, non-negative interval of $\left(2b\pi +\frac{\pi }{2},2b\pi +\pi \right)$ (let k = 0), and obtain a new sequence $X^{\left(0\right)}$:

(A2) $$X^{\left(0\right)}=\left\{x^{\left(0\right)}\left(1\right),x^{\left(0\right)}\left(2\right),\cdots ,x^{\left(0\right)}\left(k\right),k=1,2,3,\cdots ,n\right\}$$

(3) Conduct functional transformation on sequence $X^{\left(0\right)}$: $y^{\left(0\right)}\left(k\right)=sinx^{\left(0\right)}\left(k\right)$, and obtain a data sequence of $y^{\left(0\right)}$:

(A3) $$Y^{\left(0\right)}=\left\{y^{\left(0\right)}\left(1\right),y^{\left(0\right)}\left(2\right),\cdots ,y^{\left(0\right)}\left(k\right),k=1,2,3,\cdots ,n\right\}$$

(4) Generate $Y^{\left(1\right)}$ by Accumulated Generating Operation (AGO) on data sequence (3):

(A4) $$Y^{\left(1\right)}=\left\{y^{\left(1\right)}\left(1\right),y^{\left(1\right)}\left(2\right),\cdots ,y^{\left(1\right)}\left(k\right),k=1,2,3,\cdots ,n\right\}$$

in which:

(A5) $$Y^{\left(1\right)}\left(k\right)=\overset{k}{\underset{i=1}{\sum }}y^{\left(0\right)}\left(i\right)$$

(5) Generate the immediate average of $Y^{\left(1\right)}$. Let $z^{\left(1\right)}\left(k\right)=0.5y^{\left(1\right)}\left(k\right)+y^{\left(1\right)}\left(k-1\right)$, and we can get:

(A6) $$Z^{\left(1\right)}=\left\{z^{\left(1\right)}\left(1\right),z^{\left(1\right)}\left(2\right),\cdots ,z^{\left(1\right)}\left(n\right)\right\}$$

(6) Construct the GM(1,1) Model based on sequence Y^(1)^ generated by Accumulated Generating Operation (AGO). The corresponding Albino Differential Equation can be written as:

(A7) $$\frac{dy^{\left(1\right)}\left(t\right)}{dt}+ay^{\left(1\right)}\left(t\right)=b$$

(7) Let the starting conditions be $y^{\left(1\right)}\left(1\right)=y^{\left(0\right)}\left(1\right)$, and the discrete solution of the above equation can be written as:

(A8) $${\hat{y}}^{\left(1\right)}\left(k\right)=\left(y^{\left(0\right)}\left(1\right)-\frac{b}{a}\right)e^{-ak}+\frac{b}{a}\left(k=1,2,\cdots ,n\right)$$

(8) Determine the parameter column $\beta ={\left(a,b\right)}^{T}$ by using the Least Square Method:

(A9) $$\beta ={\left(B^{T}B\right)}^{-1}B^{T}Y$$

in which:

(A10) $$B=[\begin{array}{c} -z^{\left(1\right)}\left(2\right)\;1\\ -z^{\left(1\right)}\left(3\right)\;1\\ \begin{array}{c} \cdots \\ -z^{\left(1\right)}\left(n\right)\;1 \end{array} \end{array},\;Y=[\begin{array}{c} z^{\left(0\right)}\left(2\right)\\ z^{\left(0\right)}\left(3\right)\\ \begin{array}{c} \cdots \\ z^{\left(0\right)}\left(4\right) \end{array} \end{array}$$

(9) Substituting k=1,2,⋯,n into the above equation, and we can obtain the simulated value of $Y^{\left(1\right)}$ written as ${\hat{y}}^{\left(1\right)}$:

(A11) $${\hat{y}}^{\left(1\right)}=\left\{{\hat{y}}^{\left(1\right)}\left(1\right),{\hat{y}}^{\left(1\right)}\left(2\right),\cdots ,{\hat{y}}^{\left(1\right)}\left(n\right)\right\}$$

(10) Restore to find the simulated value of $Y^{\left(0\right)}$ (written as ${\hat{y}}^{\left(0\right)}$). From ${\hat{y}}^{\left(0\right)}\left(k\right)={\hat{y}}^{\left(1\right)}\left(k\right)-{\hat{y}}^{\left(1\right)}\left(k-1\right)$, we can get:

(A12) $${\hat{y}}^{\left(0\right)}=\left\{{\hat{y}}^{\left(0\right)}\left(1\right),{\hat{y}}^{\left(0\right)}\left(2\right),\cdots ,{\hat{y}}^{\left(0\right)}\left(n\right)\right\}$$

(11) By inverse function transformation ${\hat{x}}^{\left(0\right)}\left(k\right)=\pi -arcsin{\hat{y}}^{\left(0\right)}\left(k\right)\left(let\;k=0\right)$, we can get:

(A13) $${\hat{x}}^{\left(0\right)}=\left\{{\hat{x}}^{\left(0\right)}\left(1\right),{\hat{x}}^{\left(0\right)}\left(2\right),\cdots ,{\hat{x}}^{\left(0\right)}\left(n\right)\right\}$$

(12) After data transformation and reduction, we can obtain the simulated value ${\hat{A}}^{\left(0\right)}$ of the raw data:

(A14) $${\hat{A}}^{\left(0\right)}=\left\{{\hat{a}}^{\left(0\right)}\left(1\right),{\hat{a}}^{\left(0\right)}\left(2\right),\cdots ,{\hat{a}}^{\left(0\right)}\left(n\right)\right\}$$

### Appendix B.2. MATLAB Algorithm

**Algorithm A2**
1: function [ simulation,params ] = GM( org ) 2: n=length(org); 3: for i=1:n 4: acc(i)=sum(org(1:i)); 5: end 6: for i=1:(n-1) 7: zk(i)=0.5*(acc(i)+acc(i+1)); 8: end 9: params=polyfit(zk,org(2:end),1); 10: for i=1:n 11: if i==1 12: simulation(i)=org(1); 13: else 14: simulation(i)=(org(1)+params(2)/params(1))*(1-exp(-params(1)))*exp(params(1)*(i-1)); 15: end 16: end

## Appendix C. Simulated Air Quality Assessment Score of Cities in Shandong Province

**Table A1:** Simulated air quality assessment score of cities in Shandong province in absence of pollution control policies (Jul 2018–Mar 2019).

	Jun 2018	Jul 2018	Aug 2018	Sep 2018	Oct 2018	Nov 2018	Dec 2018	Jan 2019	Feb 2019	Mar 2019
**Binzhou**	0.4693	0.4087	0.3901	0.5708	0.5293	0.4681	0.6887	0.6627	0.5836	0.5903
**Dezhou**	0.2013	0.3376	0.4571	0.3595	0.2254	0.2641	0.3837	0.1658	0.3214	0.3942
**Dongying**	0.3543	0.3943	0.5241	0.3538	0.3501	0.4872	0.5333	0.4162	0.3523	0.4207
**Heze**	0.3207	0.3829	0.5475	0.1783	0.3386	0.3023	0.1409	0.2763	0.3484	0.2150
**Ji’nan**	0.3586	0.3928	0.3690	0.3387	0.4521	0.3550	0.4107	0.4240	0.3538	0.4126
**Jining**	0.4871	0.4899	0.6039	0.3588	0.6515	0.3547	0.5456	0.6284	0.4155	0.4399
**Laiwu**	0.3737	0.4942	0.5053	0.4979	0.4438	0.4314	0.3451	0.4077	0.3154	0.2502
**Liaocheng**	0.3803	0.4917	0.5843	0.3893	0.3726	0.3299	0.3754	0.3857	0.3042	0.4044
**Linyi**	0.4217	0.3440	0.4687	0.4049	0.7218	0.3808	0.6299	0.6501	0.5548	0.6228
**Qingdao**	0.6580	0.7138	0.6337	0.7377	0.5527	0.4826	0.8180	0.9939	0.8206	0.7000
**Rizhao**	0.6147	0.6744	0.5573	0.6848	0.5836	0.6378	0.6274	0.6637	0.7036	0.6249
**Tai’an**	0.5674	0.5356	0.6245	0.5205	0.2254	0.2553	0.1306	0.2222	0.1732	0.4037
**Weifang**	0.4657	0.4466	0.5136	0.5028	0.6052	0.6527	0.4807	0.5685	0.7930	0.7436
**Weihai**	0.7515	0.8467	0.7412	0.8997	0.8635	0.8474	0.7387	0.8499	0.9644	0.8239
**Yantai**	0.8132	0.7841	0.7429	0.8346	0.8399	0.8155	0.9082	0.6351	0.7018	0.6863
**Zaozhuang**	0.4587	0.3279	0.3220	0.3757	0.3907	0.2846	0.5219	0.6180	0.5079	0.4258
**Zibo**	0.2688	0.1995	0.2174	0.1901	0.1772	0.1673	0.1279	0.1796	0.1513	0.1943

**Table A2:** Simulated air quality assessment score of cities in Shandong province in absence of pollution control policies (Apr 2019–Jan 2020).

	Apr 2019	May 2019	Jun 2019	Jul 2019	Aug 2019	Sep 2019	Oct 2019	Nov 2019	Dec 2019	Jan 2020
**Binzhou**	0.4589	0.5970	0.5032	0.4879	0.4715	0.5057	0.5329	0.5411	0.5780	0.5900
**Dezhou**	0.2506	0.2166	0.4918	0.1733	0.1384	0.4225	0.1361	0.1722	0.3034	0.3794
**Dongying**	0.3404	0.3995	0.3232	0.4061	0.3611	0.4160	0.3694	0.3493	0.3433	0.3412
**Heze**	0.4677	0.4035	0.2828	0.4831	0.3402	0.2375	0.4833	0.2330	0.3335	0.5365
**Ji’nan**	0.3753	0.4154	0.3365	0.3968	0.2565	0.2012	0.3111	0.2619	0.3101	0.3247
**Jining**	0.8452	0.2900	0.6175	0.6424	0.4057	0.2567	0.9835	0.2170	0.6088	0.6857
**Laiwu**	0.2597	0.3059	0.2650	0.4710	0.4434	0.5076	0.6774	0.6700	0.3806	0.4940
**Liaocheng**	0.3765	0.3497	0.4043	0.3825	0.2723	0.2931	0.3690	0.4266	0.4389	0.4113
**Linyi**	0.6411	0.6830	0.5715	0.6991	0.8670	0.6774	0.6348	0.6976	0.6365	0.6109
**Qingdao**	0.6665	0.6705	0.7587	0.8717	0.8922	0.8331	0.6545	0.5547	0.5007	0.6555
**Rizhao**	0.6904	0.7306	0.6246	0.7123	0.6392	0.5966	0.6737	0.6200	0.6357	0.6299
**Tai’an**	0.5227	0.3595	0.5134	0.2212	0.2896	0.0731	0.2050	0.2707	0.2362	0.3137
**Weifang**	0.4954	0.7039	0.7164	0.5883	0.4738	0.7190	0.7649	0.5166	0.6689	0.7308
**Weihai**	0.7300	0.8702	0.8472	0.8055	0.6969	0.7605	0.7971	0.8820	0.8256	0.8628
**Yantai**	0.7975	0.9157	0.8078	0.8482	0.7866	0.8289	0.8959	0.7600	0.8909	0.8166
**Zaozhuang**	0.4850	0.3766	0.3950	0.4846	0.5638	0.4707	0.5393	0.5008	0.2515	0.2925
**Zibo**	0.1961	0.1328	0.1242	0.1509	0.1189	0.1656	0.1555	0.1916	0.1762	0.1229

## Appendix D. Air Quality Assessment Score of Cities in Shandong Province

**Table A3:** Air quality assessment score of cities in Shandong province (2017.02–2017.10).

	Feb 2017	Mar 2017	Apr 2017	May 2017	Jun 2017	Jul 2017	Aug 2017	Sep 2017	Oct 2017
**Binzhou**	0.4693	0.4087	0.3901	0.5708	0.4726	0.5566	0.7403	0.6251	0.5028
**Dezhou**	0.2018	0.3376	0.4571	0.3595	0.2572	0.3887	0.1861	0.2370	0.3223
**Dongying**	0.6043	0.3943	0.5241	0.3938	0.3617	0.4957	0.2926	0.3324	0.4224
**Heze**	0.3207	0.3829	0.5475	0.1783	0.3884	0.5523	0.5983	0.4373	0.3112
**Ji’nan**	0.3586	0.3928	0.3690	0.3387	0.3639	0.3235	0.5826	0.3440	0.3813
**Jining**	0.4871	0.4899	0.6039	0.3588	0.3807	0.5662	0.5822	0.3576	0.2815
**Laiwu**	0.3737	0.4942	0.5053	0.4979	0.4686	0.3778	0.5765	0.4331	0.3782
**Liaocheng**	0.3803	0.4917	0.5843	0.3893	0.4317	0.4499	0.4604	0.3505	0.2772
**Linyi**	0.4217	0.3440	0.4687	0.4049	0.3210	0.5851	0.5420	0.4476	0.3071
**Qingdao**	0.6580	0.7138	0.6337	0.7377	0.6815	0.8423	0.8409	0.7980	0.7284
**Rizhao**	0.6147	0.6744	0.5573	0.6848	0.7229	0.7726	0.7831	0.5778	0.5376
**Tai’an**	0.5674	0.5356	0.6245	0.5205	0.5762	0.5006	0.4991	0.5847	0.5268
**Weifang**	0.4657	0.4466	0.5136	0.5028	0.4645	0.6205	0.4024	0.4219	0.4576
**Weihai**	0.7515	0.8467	0.7412	0.8997	0.9603	0.9960	0.7977	0.8049	0.8659
**Yantai**	0.8132	0.7841	0.7429	0.8346	0.8554	0.9336	0.7846	0.7722	0.8368
**Zaozhuang**	0.4587	0.3279	0.3220	0.3757	0.5635	0.6346	0.5767	0.4136	0.3343
**Zibo**	0.2688	0.1995	0.2174	0.1901	0.1423	0.1924	0.1511	0.1228	0.2080

**Table A4:** Air quality assessment score of cities in Shandong province (2017.11–2018.07).

	Nov 2017	Dec 2017	Jan 2018	Feb 2018	Mar 2018	Apr 2018	May 2018	Jun 2018	Jul 2018
**Binzhou**	0.5048	0.4956	0.5646	0.5743	0.3737	0.6929	0.5513	0.5203	0.3093
**Dezhou**	0.2880	0.2075	0.3567	0.4425	0.2110	0.4540	0.2826	0.2540	0.1563
**Dongying**	0.5017	0.4729	0.6229	0.6313	0.4724	0.4014	0.3601	0.4233	0.3276
**Heze**	0.3718	0.2586	0.1786	0.2782	0.1957	0.3086	0.4703	0.3088	0.3965
**Ji’nan**	0.3393	0.3516	0.2747	0.5103	0.3588	0.3982	0.2939	0.3153	0.2662
**Jining**	0.3936	0.3680	0.2704	0.4177	0.4782	0.6234	0.5742	0.5007	0.5403
**Laiwu**	0.4420	0.4546	0.3534	0.4141	0.3595	0.2827	0.2887	0.3245	0.3769
**Liaocheng**	0.4307	0.2807	0.3294	0.4870	0.2247	0.4418	0.3591	0.3264	0.2706
**Linyi**	0.5662	0.4880	0.3317	0.5256	0.4303	0.4773	0.5860	0.6756	0.6046
**Qingdao**	0.6451	0.6550	0.6536	0.7268	0.8332	0.6738	0.7608	0.8819	0.9198
**Rizhao**	0.6568	0.5815	0.5373	0.6562	0.6845	0.7263	0.5074	0.6835	0.7604
**Tai’an**	0.5946	0.5635	0.3728	0.4538	0.5123	0.5392	0.5145	0.3303	0.4370
**Weifang**	0.5278	0.5103	0.5513	0.5938	0.5861	0.5769	0.3934	0.5158	0.6231
**Weihai**	0.7928	0.8931	0.9086	0.9255	0.9573	0.7429	0.7758	0.9246	0.9455
**Yantai**	0.7557	0.8579	0.8280	0.8420	0.9072	0.8239	0.7863	0.7946	0.8557
**Zaozhuang**	0.4012	0.5356	0.4352	0.4335	0.3401	0.5323	0.6265	0.5597	0.6173
**Zibo**	0.4050	0.2179	0.3381	0.4189	0.3264	0.3476	0.1386	0.1222	0.2212

**Table A5:** Air quality assessment score of cities in Shandong province (2018.08–2019.04).

	Aug 2018	Sep 2018	Oct 2018	Nov 2018	Dec 2018	Jan 2019	Feb 2019	Mar 2019	Apr 2019
**Binzhou**	0.4968	0.3968	0.3879	0.2849	0.3336	0.3344	0.4514	0.4211	0.6073
**Dezhou**	0.3763	0.2174	0.2221	0.2641	0.3231	0.3863	0.2223	0.4151	0.3470
**Dongying**	0.4025	0.3901	0.2877	0.3165	0.4355	0.4877	0.4231	0.3153	0.3410
**Heze**	0.4425	0.2642	0.4495	0.3528	0.2390	0.3814	0.2147	0.2606	0.4884
**Ji’nan**	0.3413	0.2644	0.3250	0.4874	0.3234	0.2387	0.3490	0.4094	0.3334
**Jining**	0.3829	0.3822	0.6302	0.5571	0.2882	0.4977	0.4199	0.5494	0.6756
**Laiwu**	0.4487	0.3563	0.4516	0.3964	0.3792	0.3871	0.5534	0.3937	0.4579
**Liaocheng**	0.3337	0.2336	0.3741	0.3846	0.2595	0.3647	0.2222	0.3246	0.3481
**Linyi**	0.4829	0.4700	0.6464	0.4127	0.4466	0.5109	0.3255	0.4592	0.4308
**Qingdao**	0.8541	0.6663	0.7058	0.6411	0.6749	0.6227	0.6105	0.7959	0.7534
**Rizhao**	0.5868	0.5914	0.7569	0.4791	0.6594	0.6059	0.5738	0.7253	0.7740
**Tai’an**	0.3587	0.3126	0.5718	0.4012	0.4692	0.4701	0.4507	0.5208	0.7365
**Weifang**	0.5582	0.4674	0.4091	0.3424	0.4797	0.4790	0.3865	0.5247	0.6038
**Weihai**	0.8148	0.9052	0.9523	0.8653	0.9769	0.9098	0.8544	0.9254	0.9027
**Yantai**	0.7871	0.7545	0.7382	0.5768	0.7291	0.6860	0.6614	0.6192	0.7176
**Zaozhuang**	0.5393	0.4999	0.5435	0.4219	0.5339	0.4843	0.3758	0.5245	0.4168
**Zibo**	0.2076	0.1306	0.2313	0.3370	0.1681	0.2232	0.3051	0.1737	0.2288

**Table A6:** Air quality assessment score of cities in Shandong province (2019.05–2020.01).

	May 2019	Jun 2019	Jul 2019	Aug 2019	Sep 2019	Oct 2019	Nov 2019	Dec 2019	Jan 2020
**Binzhou**	0.3714	0.3612	0.4115	0.3523	0.2650	0.3756	0.3453	0.4283	0.5453
**Dezhou**	0.2484	0.3071	0.3168	0.2639	0.4060	0.3268	0.3626	0.4559	0.3030
**Dongying**	0.4403	0.2484	0.4439	0.5096	0.4844	0.3944	0.4287	0.4638	0.5180
**Heze**	0.3248	0.3426	0.6363	0.4881	0.4564	0.3525	0.2068	0.1986	0.3595
**Ji’nan**	0.5062	0.2908	0.2883	0.3457	0.2771	0.4600	0.4400	0.3530	0.4027
**Jining**	0.3684	0.3473	0.5284	0.2307	0.3699	0.4314	0.4236	0.3621	0.3749
**Laiwu**	0.4134	0.4161	0.6074	0.3315	0.4500	0.4248	0.2239	0.2789	0.2923
**Liaocheng**	0.4019	0.4862	0.5510	0.4417	0.3671	0.5213	0.3599	0.3860	0.4964
**Linyi**	0.3617	0.3595	0.4725	0.4233	0.6837	0.3872	0.2622	0.3583	0.3022
**Qingdao**	0.7964	0.8410	0.7258	0.7230	0.6215	0.5311	0.6265	0.6072	0.6464
**Rizhao**	0.6808	0.8293	0.7780	0.6817	0.7439	0.6231	0.5386	0.5888	0.5628
**Tai’an**	0.5209	0.3463	0.5295	0.5100	0.4930	0.5573	0.4499	0.4335	0.3706
**Weifang**	0.6298	0.4992	0.5548	0.5575	0.4844	0.3707	0.3471	0.4120	0.3854
**Weihai**	0.9306	0.9091	0.8622	0.9209	0.7558	0.8771	0.9131	0.8961	0.8767
**Yantai**	0.8541	0.7482	0.7645	0.8779	0.6741	0.6912	0.7836	0.7778	0.7557
**Zaozhuang**	0.4496	0.4557	0.6394	0.4910	0.8135	0.3014	0.2314	0.3097	0.2534
**Zibo**	0.3701	0.1117	0.2583	0.0539	0.2607	0.2528	0.2210	0.2340	0.1633

## Appendix E. The Error Report of the Model

**Table A7:** Air quality assessment score of cities in Shandong province (2018.02–2018.05).

	Feb 2018		Mar 2018		Apr 2018		May 2018	
	Real	Simulated	Real	Simulated	Real	Simulated	Real	Simulated
**Binzhou**	0.5743	0.5667	0.3737	0.3600	0.6929	0.6790	0.5513	0.5293
**Dezhou**	0.4425	0.4278	0.2110	0.2026	0.4540	0.4449	0.2826	0.2732
**Dongying**	0.6313	0.6292	0.4724	0.4646	0.4014	0.4000	0.3601	0.3505
**Heze**	0.2782	0.2717	0.1957	0.1891	0.3086	0.3055	0.4703	0.4625
**Ji’nan**	0.5103	0.5086	0.3588	0.3492	0.3982	0.3849	0.2939	0.2920
**Jining**	0.4177	0.4079	0.4782	0.4703	0.6234	0.6089	0.5742	0.5512
**Laiwu**	0.4141	0.4127	0.3595	0.3475	0.2827	0.2713	0.2887	0.2867
**Liaocheng**	0.4870	0.4821	0.2247	0.2150	0.4418	0.4285	0.3591	0.3507
**Linyi**	0.5256	0.5116	0.4303	0.4203	0.4773	0.4598	0.5860	0.5860
**Qingdao**	0.7268	0.6929	0.8332	0.8165	0.6738	0.6559	0.7608	0.7456
**Rizhao**	0.6562	0.6562	0.6845	0.6685	0.7263	0.7166	0.5074	0.4855
**Tai’an**	0.4538	0.4326	0.5123	0.5089	0.5392	0.5230	0.5145	0.5025
**Weifang**	0.5938	0.5918	0.5861	0.5607	0.5769	0.5653	0.3934	0.3855
**Weihai**	0.9255	0.9224	0.9573	0.9542	0.7429	0.7231	0.7758	0.7396
**Yantai**	0.8420	0.8279	0.9072	0.8951	0.8239	0.8157	0.7863	0.7784
**Zaozhuang**	0.4335	0.4335	0.3401	0.3390	0.5323	0.5181	0.6265	0.6014
**Zibo**	0.4189	0.4134	0.3264	0.3122	0.3476	0.3325	0.1386	0.1353

**Table A8:** The error between real assessment score core and simulated score (2018.02–2018.05).

	Error (%)				Mean Error (%)
	Feb 2018	Mar 2018	Apr 2018	May 2018	
**Binzhou**	1.33	3.67	2.00	4.00	2.75
**Dezhou**	3.33	4.00	2.00	3.33	3.17
**Dongying**	0.33	1.67	0.33	2.67	1.25
**Heze**	2.33	3.33	1.00	1.67	2.08
**Ji’nan**	0.33	2.68	3.33	0.67	1.75
**Jining**	2.33	1.67	2.33	4.00	2.58
**Laiwu**	0.33	3.33	4.00	0.67	2.08
**Liaocheng**	1.00	4.33	3.00	2.33	2.67
**Linyi**	2.67	2.33	3.67	0.00	2.17
**Qingdao**	4.67	2.00	2.67	2.00	2.83
**Rizhao**	0.00	2.33	1.33	4.33	2.00
**Tai’an**	4.67	0.67	3.00	2.33	2.67
**Weifang**	0.33	4.33	2.00	2.00	2.17
**Weihai**	0.33	0.33	2.67	4.67	2.00
**Yantai**	1.67	1.33	1.00	1.00	1.25
**Zaozhuang**	0.00	0.33	2.67	4.00	1.75
**Zibo**	1.33	4.33	4.33	2.33	3.08
